# Nasopharyngeal Pneumococcal Colonization Density Is Associated With Severe Pneumonia in Young Children in the Lao People’s Democratic Republic

**DOI:** 10.1093/infdis/jiab239

**Published:** 2021-05-11

**Authors:** Olivia J J Carr, Keoudomphone Vilivong, Laddaphone Bounvilay, Eileen M Dunne, Jana Y R Lai, Jocelyn Chan, Malisa Vongsakid, Anisone Changthongthip, C Siladeth, Belinda Ortika, Cattram Nguyen, Mayfong Mayxay, Paul N Newton, Kim Mulholland, Lien A H Do, Audrey Dubot-Pérès, Catherine Satzke, David A B Dance, Fiona M Russell

**Affiliations:** 1 University of Tasmania, Hobart, Tasmania, Australia; 2 Murdoch Children’s Research Institute, Melbourne, Victoria, Australia; 3 Lao-Oxford-Mahosot Hospital-Wellcome Trust Research Unit, Microbiology Laboratory, Mahosot Hospital, Vientiane, Lao People’s Democratic Republic; 4 World Health Organization, Lao People’s Democratic Republic; 5 Department of Paediatrics, University of Melbourne, Parkville, Victoria, Australia; 6 Institute of Research and Education Development, University of Health Sciences, Ministry of Health, Vientiane, Lao People’s Democratic Republic; 7 Centre for Tropical Medicine and Global Health, Nuffield Department of Clinical Medicine, University of Oxford, Oxford, United Kingdom; 8 Faculty of Infectious and Tropical Diseases, London School of Hygiene and Tropical Medicine, London, United Kingdom; 9 Department of Infectious Disease Epidemiology, London School of Hygiene and Tropical Medicine, London, United Kingdom; 10 Unité des Virus Émergents (UVE: Aix-Marseille Univ-Institut de Recherche pour le Développement 190-Inserm 1207), Marseille, France; 11 Department of Microbiology and Immunology, Peter Doherty Institute for Infection and Immunity, University of Melbourne, Parkville, Australia

**Keywords:** pneumococcus, pneumonia, density, nasopharynx

## Abstract

**Background:**

No studies have explored the association between pneumococcal nasopharyngeal density and severe pneumonia using the World Health Organization (WHO) 2013 definition. In Lao People’s Democratic Republic (Lao PDR), we determine the association between nasopharyngeal pneumococcal density and severe pneumonia in children.

**Methods:**

A prospective observational study was undertaken at Mahosot Hospital, Vientiane, from 2014 to mid-2018. Children <5 years admitted with acute respiratory infections (ARIs) were included. Clinical and demographic data were collected alongside nasopharyngeal swabs for pneumococcal quantification by *lytA* real-time quantitative polymerase chain reaction. Severe pneumonia was defined using the 2013 WHO definition. For pneumococcal carriers, a logistic regression model examined the association between pneumococcal density and severe pneumonia, after adjusting for potential confounders including demographic and household factors, 13-valent pneumococcal conjugate vaccine status, respiratory syncytial virus co-detection, and preadmission antibiotics.

**Results:**

Of 1268 participants with ARI, 32.3% (n = 410) had severe pneumonia and 36.9% (n = 468) had pneumococcal carriage. For pneumococcal carriers, pneumococcal density was positively associated with severe pneumonia (adjusted odds ratio, 1.4 [95% confidence interval, 1.1–1.8]; *P* = .020).

**Conclusions:**

Among children with ARIs and pneumococcal carriage, pneumococcal carriage density was positively associated with severe pneumonia in Lao PDR. Further studies may determine if pneumococcal density is a useful marker for pneumococcal conjugate vaccine impact on childhood pneumonia.

## Teaser

No studies have explored the association between pneumococcal nasopharyngeal density and severe pneumonia using the World Health Organization 2013 definition. Among children with acute respiratory infections and pneumococcal carriage, pneumococcal carriage density was positively associated with severe pneumonia in Laos.

*Streptococcus pneumoniae* (pneumococcus) is a major cause of childhood mortality globally [1]. It is a leading cause of bacterial pneumonia in young children, resulting in an estimated 294 000 deaths worldwide in children <5 years of age [1, 2]. However, it is difficult to identify the etiology of pneumonia, as there is no noninvasive way to directly examine the lung in children.

Colonization with the pneumococcus is common in young children, and although usually asymptomatic, it remains a necessary precursor to invasive pneumococcal disease [3–5]. The role of pneumococcal carriage in the epidemiology of pneumonia is still being defined [6]. Some studies conducted in children have found that the density of pneumococcus in the nasopharynx is positively associated with pneumonia using a variety of definitions including radiologically confirmed pneumonia, very severe pneumonia, microbiologically confirmed pneumonia, and hypoxic pneumonia [7–10] as there is no gold-standard pneumonia definition. Furthermore, as many low- and middle-income countries (LMICs) are unable to perform radiographs and do not have adequate microbiology services, finding an association between pneumococcal density and the current (2013) clinical World Health Organization (WHO) severe pneumonia definition is important for these settings as, to our knowledge, no studies have explored this association [11].

The Lao People’s Democratic Republic (Lao PDR) is an LMIC in Southeast Asia with a population of approximately 7 million people. The infant mortality rate is 40 per 1000 live births [12]. Each year, approximately 14 000 deaths occur in children <5 years of age, with an estimated 18% of deaths due to pneumonia [13]. The aim of this study is to describe the association between pneumococcal nasopharyngeal density and severe pneumonia, as defined by WHO [11], in hospitalized children in Lao PDR.

## MATERIALS AND METHODS

We undertook a prospective hospital-based observational study of acute respiratory infection (ARI) in children aged 2–59 months admitted to Mahosot Hospital [14] in Vientiane, the capital of Lao PDR, where 13% of the population lives. Mahosot Hospital is a tertiary hospital that admits both adults and children. During the study period, it had a capacity of 365 beds, of which 47 were for pediatric patients.

Potential participants were identified from pediatric intensive care and the pediatric infectious diseases and general pediatrics wards. Data were collected as part of the Pneumococcal Carriage in Pneumonia to Investigate Vaccine Effects (PneuCAPTIVE) study, which commenced in December 2013 [14]. The analysis presented here includes data collected between December 2013 to June 2018, for which both pneumococcal carriage and respiratory syncytial virus (RSV) testing results were available, as children with coinfection with respiratory viruses tend to have high nasopharyngeal pneumococcal densities so RSV was considered an important confounder [8].

### Study Procedures

Following informed consent by the parents/guardians, children aged 2–59 months were enrolled in the study based on the following eligibility criteria: age, a history of illness ≤14 days with documented tympanic fever (temperature >38.0°C), and 1 or more of dyspnea (as described by patient or caregiver and/or study doctor), cough, rhinitis, and/or abnormal auscultatory examination.

Parents/guardians were interviewed by study staff who recorded the following demographic and household information: age, sex, ethnicity (Lao Loum or other minority groups), residential location (rural or urban/periurban), number of children <5 years of age, presence of a cigarette smoker in the house, the type of indoor cooking fuel used (coal, wood, gas, or other), monthly household income, and parent-reported antibiotic use in the week prior to admission. For clinical signs and symptoms relating to severe pneumonia, study doctors extracted data from the patients’ medical records at the time of admission as recorded by the treating doctor. Based on these signs and symptoms, the study doctor reclassified severe pneumonia according to the 2013 WHO definition: cough and/or difficulty breathing and tachypnea (≥50 breaths per minute for children aged 2–11 months and ≥40 breaths per minute for children aged 12–59 months) with any 1 of oxygen saturation <90%, central cyanosis, severe respiratory distress, inability to drink or breastfeed or vomiting everything, altered consciousness, and convulsions [11]. Participants who did not have severe pneumonia were categorized as having “other ARI.” Vaccination with the 13-valent pneumococcal conjugate vaccine (PCV13) was initiated in November 2013 in Lao PDR, with doses administered at 6, 10, and 14 weeks of age. PCV13 vaccination status for each participant was recorded from either health center immunization registers or direct visualization of parent-held child health cards. Vaccinated was defined as any infant aged <12 months who had received ≥2 doses of PCV13 or children ≥12 months who had received ≥1 dose of PCV13. Undervaccinated was defined as any child who did not meet these requirements [15].

Nasopharyngeal swabs were collected from participants using pediatric flocked swabs (Copan Diagnostics) according to WHO guidelines [16]. The swabs were collected by the study staff each morning Monday to Friday. Therefore, the majority were collected within 24 hours of admission, except for those admitted over the weekend. After collection, the swab was placed directly in a sterile cryovial of 1.0 mL skim milk, tryptone, glucose, and glycerol (STGG) medium. The samples were stored at –80°C [16], and then transported to the Murdoch Children’s Research Institute (Parkville, Australia) on dry ice for testing. DNA was extracted from 100 μL of STGG medium (following enzymatic treatment) using a MagNA Pure LC machine (Roche) using the DNA Isolation Kit III (bacteria, fungi) (Roche) as previously described [16]. Pneumococci were detected by real-time quantitative polymerase chain reaction (qPCR) targeting the *lytA* gene [17]. Following *lytA* qPCR, the bacterial density (reported as genome equivalents [ge]/mL) was estimated by reference to a standard curve of pneumococcal DNA with the assumption that each pneumococcal cell contains one 2-Mb genome, and each genome contains a single copy of the *lytA* gene [17]. Samples were considered to contain pneumococci if they were *lytA* positive, or *lytA* equivocal with a serotype detected by microarray.

For RSV testing, methods have been described elsewhere [18]. In brief, oral and nasal swabs were collected at the same time from each participant, and the specimens were placed in Virocult vials containing viral transport medium (Sigma Virocult, MWE) and transported to the laboratory [18]. Swabs were then unloaded from the vial, 100 μL of fluid from both the oral and nasal swabs was combined, and nucleic acids were extracted via the Cador Pathogen 96 QIAcube HT kit (Qiagen), with an elution volume of 90 μL. Samples collected from December 2013 to December 2014 were assessed for the presence of RSV via the Fast-track Diagnostics respiratory pathogens 33 kit. This consisted of multiplexed probe based real-time PCRs for the detection of the pathogens. Samples collected after December 2014 were tested by probe-based real-time RT-PCR using the iTaq Universal Probes One-Step reverse transcriptase kit (Bio-Rad) and primers/probe as previously described [19], from 10 µL of nucleic acids, in a final reaction volume of 30 µL. A sample was considered positive for RSV if the real-time PCR assay had a cycle threshold value of <35.

### Statistical Analysis

Data relating to clinical and demographic factors were double entered into a REDCap (https://redcap.vanderbilt.edu/) database. Laboratory data were imported from Excel (Microsoft Office) to Stata version 15.1 [20] and cleaned prior to merging with the cleaned demographic and clinical data.

Participant characteristics were stratified by severity of illness (severe pneumonia and ARI) and summarized by counts and percentages. For pneumococcal carriers, pneumococcal density was log_10_ transformed and reported as median log_10_ ge/mL with interquartile range (IQR). Wilcoxon signed-rank test was used to compare continuous variables. Pearson χ ^2^ test was used for categorical variables, and 1-way analysis of variance was used for continuous variables across >2 groups.

To determine the association between pneumococcal density and severe pneumonia, logistic regression was used to model severe pneumonia as a function of pneumococcal carriage density and other covariates, reported as odds ratios (ORs) and 95% confidence intervals (CIs). Only pneumococcal carriers and those with complete data were included in this analysis. To assess for potential collinearity between covariates, correlation between potential confounders was determined using Pearson *r*. The multivariable model was built using variables found to be significant at the univariable level (*P* < .2) and those identified a priori. Variables identified a priori were visualized using a directed acyclic graph (DAG) (http://www.dagitty.net/) and included all possible variables that were related to severe pneumonia and were potential confounders (Supplementary Figure 1). The DAG and the univariable analyses identified the following variables for inclusion in the multivariable model: age, ethnicity, residential location, other children <5 years of age at home, cigarette smoker in house, below the poverty line (defined according to the World Bank as <1.25 US dollars [USD] per day [2013–2014] and <1.9 USD per day [2015–2018]) [21], pneumococcal density (continuous variable), PCV13 vaccination status, coinfection with RSV, and prior administration of antibiotics. The final multivariable model was considered to fit the data well if the Hosmer–Lemeshow goodness-of-fit statistic was >0.05.

This study was conducted according to the protocol approved by The Royal Children’s Hospital Melbourne (human research ethics committee [HREC] number 33177B; Murdoch Children’s Research Institute); Oxford Tropical Research Ethics Committee (1050-13; Lao-Oxford-Mahosot Hospital–Wellcome Trust Research Unit, Oxford University); Ethics Research Committee (2013.30.LAO.2.EIP; Western Pacific Regional Office of WHO); National Ethics Committee for Health Research (number 057/2013; Ministry of Health, Lao PDR); and University of Tasmania Human Research Ethics Committee (HREC number H0018186).

## RESULTS

In total, 1268 children with ARI were included in this analysis (Table 1). Participant characteristics are shown in Table 1. Of the 1268 participants, 32.3% had severe pneumonia and 36.9% were pneumococcal carriers. Children with severe pneumonia were younger than other ARI cases (median age, 11 vs 17 months), were more likely to live below the poverty line (8.8% vs 5.3%), and were more likely to have received antibiotic therapy in the week prior to admission (58.4% vs 50.5%). Of the 468 pneumococcal carriers, 125 had severe pneumonia (30.5%). There was no difference in median pneumococcal density between severe pneumonia cases (5.83 [IQR, 5.14–6.36]) and other ARIs (5.62 [IQR, 5.02–6.30]). The median duration of illness prior to admission was 4 days (IQR, 2–5 days).

**Table 1.** Characteristics of Study Participants in Children Hospitalized With Acute Respiratory Infections in Lao People’s Democratic Republic, by Pneumonia Severity (N = 1268)

Characteristics	All Participants (N = 1268)	Severe Pneumonia^a^ (n = 410)	Other ARI (n = 858)	*P* Value^b^
Demographics				
Age, mo, median (IQR)	15 (8–25)	11 (6–20)	17 (10–28)	<.001
Age group, mo				
2–5	220 (17.4)	113 (27.6)	107 (12.5)	<.001
6–11	288 (22.7)	101 (24.6)	187 (21.8)	
12–23	412 (32.5)	126 (30.7)	286 (33.3)	
24–59	348 (27.4)	70 (17.1)	278 (32.4)	
Male sex	718 (56.6)	233 (56.8)	485 (56.5)	.919
Ethnicity				
Minority group	130 (10.2)	57 (13.9)	73 (8.5)	.003
Lao Loum	1138 (89.8)	353 (86.1)	785 (91.5)	
Household features				
Residential location				
Outside Vientiane capital	1205 (95.0)	382 (93.2)	823 (95.9)	.035
Vientiane capital	63 (5.0)	28 (6.8)	35 (4.1)	
Other children aged <5 y in house	(n = 1261)	(n = 408)	(n = 853)	
0–1	822 (65.2)	253 (62.0)	569 (66.7)	.101
≥2	439 (34.8)	155 (38.0)	284 (33.3)	
Cigarette smoker in the house	(n = 1254)	(n = 404)	(n = 850)	
Yes	554 (44.2)	203 (50.3)	351 (41.3)	.003
Source of cooking fuel	(n = 960)	(n = 296)	(n = 664)	
Smoke-producing fuel	418 (43.5)	128 (43.2)	290 (43.7)	.901
Poverty				
Below poverty line^c^	81 (6.43)	36 (8.8)	45 (5.3)	.016
Clinical features				
Pneumococcal carriage	468 (36.9)	125 (30.5)	343 (40.0)	.001
Pneumococcal density^d^ (carriers only)	(n = 468)	(n = 125)	(n = 343)	
Median (IQR)	5.67 (5.02–6.32)	5.83 (5.14–6.36)	5.62 (5.02–6.30)	.097
PCV13 vaccinated^e^	(n = 1081)	(n = 354)	(n = 727)	
Undervaccinated	450 (41.6)	159 (44.9)	291 (40.0)	.126
Vaccinated	631 (58.4)	195 (55.1)	436 (60.0)	
RSV detected	(n = 1258)	(n = 380)	(n = 773)	
Yes	341 (27.1)	125 (30.9)	216 (25.3)	.039
Duration of illness prior to admission	(n = 1265)	(n = 410)	(n = 855)	
Median days (IQR)	4 (2–5)	3 (2–5)	4 (2–5)	.922
Prior antibiotic use^f^	(n = 1216)	(n = 394)	(n = 822)	
Yes	645 (53.0)	230 (58.4)	415 (50.5)	.010

Data are presented as No. (%) unless otherwise indicated.

Abbreviations: ARI, acute respiratory infection; IQR, interquartile range; PCV13, 13-valent pneumococcal conjugate vaccine; RSV, respiratory syncytial virus.

^a^World Health Organization. Pocket book of hospital care for children: guidelines for the management of common illnesses with limited resources. Severe pneumonia defined as cough and/or difficulty breathing and tachypnea (≥50 breaths per minute for children aged 2–11 months and ≥40 breaths per minute for children aged 12–59 months) with any 1 of oxygen saturation <90%, central cyanosis, severe respiratory distress, inability to drink or breastfeed or vomiting everything, altered consciousness, and convulsions.

^b^Compares ARI and severe pneumonia. χ ^2^ for categorical variables, Wilcoxon rank-sum test for continuous variables.

^c^Poverty line (World Bank) defined as <1.25 US dollars (USD) per day (2013–2014); <1.9 USD per day (2015–2018) [21].

^d^Pneumococcal density measured in log_10_ genome equivalents/mL.

^e^Vaccinated defined as infants aged <12 months who have received at least 2 doses of PCV13 or children aged ≥12 months who have received at least 1 dose of PCV13. Undervaccinated defined as any child who does not meet these requirements.

^f^Self-reported antibiotic therapy in the week before admission.

Table 2 shows the univariable and multivariable analysis of variables associated with severe pneumonia for the 372 pneumococcal carriers who had complete data for all variables in the multivariable model and were therefore included in the analysis. At the univariable level, there was some evidence of a positive association between pneumococcal density and severe pneumonia in children who carried pneumococci (OR, 1.2 [95% CI, 1.0–1.5]; *P* = .074). The youngest age group, 2–5 months, was 4.2 times more likely to have severe pneumonia than the 24- to 59-month age group (OR, 4.2 [95% CI, 2.9–6.1]; *P* < .001). Other variables strongly associated with severe pneumonia at the univariable level included being from a minority ethnic group, living in Vientiane capital, having a cigarette smoker in the house and being coinfected with RSV. When assessed for correlation, no variables were found to be strongly correlated.

**Table 2.** Associations Between Potential Confounders and Severe Pneumonia^a^ in 2- to 59-Month-Old Children Admitted to Hospital With Acute Respiratory Infections in Lao People’s Democratic Republic (n = 372)^b^

Variable	Unadjusted OR (95% CI)	*P* Value	Adjusted OR^c^ (95% CI)	*P* Value
Main variable				
Pneumococcal density (carriers only)^d^	1.2 (1.0–1.5)	.074	1.4 (1.1–1.8)	.020
Demographics				
Age, mo				
2–5	4.2 (2.9––6.1)	<.001	2.3 (1.0–5.2)	.085
6–11	2.1 (1.5–3.1)		2.6 (1.2–5.5)	
12–23	1.7 (1.3–2.5)		1.8 (.9–3.6)	
24–59	Ref		Ref	
Ethnicity				
Lao Loum	Ref		Ref	
Minority group	1.7 (1.2–2.5)	.003	1.2 (.5–2.6)	.676
Household features				
Residential location				
Outside Vientiane capital	Ref		Ref	
Vientiane capital	1.7 (1.0–2.9)	.037	1.2 (.2–6.4)	.872
Other children aged <5 y in house				
0–1	Ref		Ref	
≥2	1.2 (1.0–1.6)	.102	1.1 (.6–1.7)	.826
Cigarette smoker in the house				
No	Ref		Ref	-
Yes	1.4 (1.1–1.8)	.003	0.9 (.5–1.4)	.611
Source of cooking fuel				
Non-smoke-producing fuel	Ref		…	
Smoke-producing fuel	1.0 (.8–1.3)	.901	…	
Poverty^e^				
At/above poverty line	Ref		Ref	
Below poverty line	1.7 (1.1–2.7)	.017	1.4 (.6–3.3)	.381
Clinical features				
PCV13 vaccinated^f^				
Vaccinated	Ref		Ref	
Undervaccinated	1.2 (.9–1.6)	.126	1.5 (.9–2.6)	.135
RSV detection				
No	Ref		Ref	
Yes	1.3 (1.0–1.7)	.039	1.3 (.8–2.2)	.342
Prior antibiotic use				
Yes	Ref		Ref	
No	0.7 (.6–.9)	.010	0.9 (.5–1.5)	.640

Abbreviations: CI, confidence interval; OR, odds ratio; PCV13, 13-valent pneumococcal conjugate vaccine; Ref, reference group; RSV, respiratory syncytial virus.

^a^World Health Organization. Pocket book of hospital care for children: guidelines for the management of common illnesses with limited resources. Severe pneumonia defined as cough and/or difficulty breathing and tachypnea (≥50 breaths per minute for children aged 2–11 months and ≥40 breaths per minute for children aged 12–59 months) with any 1 of oxygen saturation <90%, central cyanosis, severe respiratory distress, inability to drink or breastfeed or vomiting everything, altered consciousness, and convulsions.

^b^Analysis only conducted on those participants with complete data available.

^c^Adjusted for age, ethnicity, residential location, other children aged <5 years at home, cigarette smoker in house, monthly income, pneumococcal carriage density, PCV13 vaccination status, and coinfection with RSV.

^d^Pneumococcal density measured in log_10_ genome equivalents/mL.

^e^Poverty line (World Bank) defined as <1.25 US dollars (USD) a day (2013–2014) or <1.9 USD a day (2015–2018) [21].

^f^Vaccinated defined as infants aged <12 months who have received at least 2 doses of PCV13 or children aged ≥12 months who have received at least 1 dose of PCV13. Undervaccinated defined as any child who does not meet these requirements.

**Figure 1.**
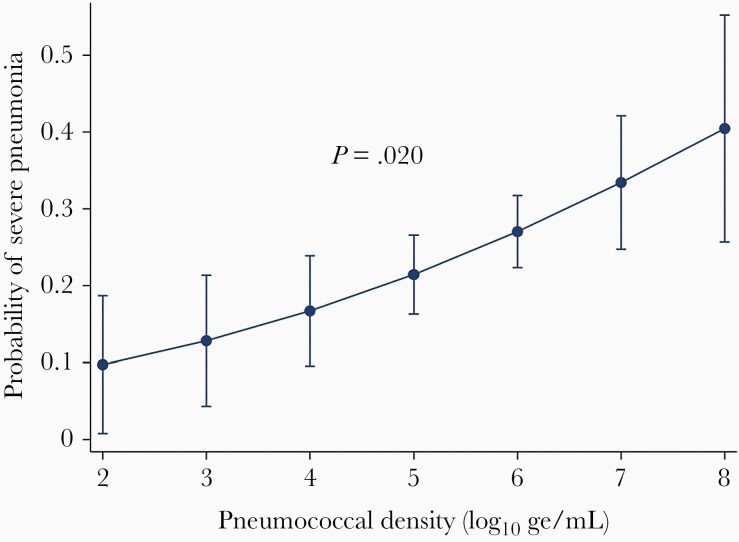
Probability of severe pneumonia [11] in children aged 2–59 months admitted to hospital with an acute respiratory infection and pneumococcal carriage, Lao People’s Democratic Republic, by pneumococcal density in log_10_ genome equivalents/mL. Severe pneumonia was defined as cough and/or difficulty breathing and tachypnea (≥50 breaths per minute for children aged 2–11 months and ≥40 breaths per minute for children aged 12–59 months) with any 1 of oxygen saturation <90%, central cyanosis, severe respiratory distress, inability to drink or breastfeed or vomiting everything, altered consciousness, and convulsions. Data adjusted for age, ethnicity, residential location, other children <5 years of age at home, cigarette smoker in house, monthly income, 13-valent pneumococcal conjugate vaccine status, coinfection with respiratory syncytial virus, and preadmission antibiotics.

Three hundred and seventy-two pneumococcal carriers were included in the analysis, of which 94 (25.3%) had severe pneumonia. Of this subset, the median age was 15 months (IQR, 9–25 months) and 217 (58.3%) were considered vaccinated using our definition. In the multivariable analysis, the probability of severe pneumonia increased with each log_10_ ge/mL increase in pneumococcal density among pneumococcal carriers (adjusted OR [aOR], 1.4 [95% CI, 1.1–1.8]; *P* = .020) (Table 2, Figure 1). The only other variables strongly positively associated with severe pneumonia after adjustment were age 2–5 months (aOR, 2.3 [95% CI, 1.0–5.2]; *P* = .044) and age 6–11 months (aOR, 2.6 [95% CI, 1.2–5.6]; *P* = .016) in comparison with the age group 24–59 months. The Hosmer–Lemeshow goodness-of-fit test statistic for this multivariable logistic regression model was 0.457. As it was >0.05, the model was considered a good fit for the data.

As not all pneumococcal carriers were included in the regression model (96/468 carriers excluded) due to missing variable data, we compared pneumococcal carriers with missing data to those with complete data, to determine if there were any systematic differences between the 2 groups (Supplementary Table 1). The 2 groups were similar; in particular, the median pneumococcal density and rates of severe pneumonia were similar for participants with complete and missing data.

## DISCUSSION

In hospitalized children in Lao PDR, we identified a positive association between increasing pneumococcal colonization density and the probability of severe pneumonia *in* pneumococcal carriers using the WHO 2013 severe pneumonia definition. To our knowledge, this is the first time this has been documented using this severe pneumonia definition. Other studies have found a positive association between pneumococcal density and other pneumonia definitions [7, 8, 22]. The Pneumonia Etiology Research for Child Health (PERCH) case-control study found a positive association between pneumococcal colonization density and children with microbiologically confirmed pneumococcal pneumonia, as well as radiographically confirmed pneumonia, very severe pneumonia, and hypoxic pneumonia [7]. Microbiologically confirmed pneumococcal pneumonia cases were defined in the PERCH study as having either a positive pneumococcal culture or PCR of blood, pleural fluid, or lung aspirate. A case-control study in Vietnam found that children with radiologically confirmed pneumonia had higher median pneumococcal densities than children with other lower respiratory tract infections [8]. In contrast, a Thai study found that pneumococcal nasopharyngeal density was not higher among children with severe pneumonia compared to controls [23]. However, this study combined oral and nasopharyngeal swabs into the same vial, which may have affected the findings. Additionally, prior receipt of antibiotics may also have reduced the median colonization density, but this and other potential confounders were not adjusted for in their analysis [23]. Subgroup analysis comparing cases that did and did not have prior antibiotic use in the Thai study found no difference in pneumococcal density between cases and controls [23]. In contrast, we found that after adjustment of the likely confounders, the probability of severe pneumonia increased with each log_10_ ge/mL increase in pneumococcal density.

In contrast, a Vietnamese study using quantitative culture-based methods to determine pneumococcal density found no association between radiologically confirmed pneumonia and pneumococcal load in the nasopharynx [24]. Comparing studies that use culture-based methods and molecular methods to measure density is likely to be problematic. Furthermore, more than two-thirds of the participants in the study received antibiotics prior to testing, which may have also contributed to the lack of association, and this was not adjusted for in the analysis [24].

Invasive lung aspirate studies are required to prove a causal link between pneumococcal colonization density and pneumococcal pneumonia, but these studies are invasive and difficult to do. A study from The Gambia found that lung and pleural aspirates were able to identify the pathogens responsible for radiologically confirmed pneumonia and found *S. pneumoniae* responsible for 25% of cases [25]. The PERCH study found that in lung and pleural aspirates, bacterial pathogens (most often *S. pneumoniae* [lung aspirate] and *Staphylococcus aureus* [pleural fluid]) were more commonly detected than viral pathogens [7]. Hence, the value of using pneumococcal density as a diagnostic assay would be if it were able to identify additional pneumococcal cases that were not radiologically or laboratory confirmed, which represents the vast majority of cases of pneumococcal pneumonia [26]. As such, PERCH found that a pneumococcal nasopharyngeal density >6.9 log_10_ copies/mL was associated with radiologically confirmed pneumonia, very severe pneumonia, and hypoxic pneumonia, but performance varied by site. Pneumococcal density improved the detection of pneumococcal pneumonia over blood culture confirmation, which is highly specific but insensitive, and is only available in settings with good microbiology services. However, the PERCH authors concluded that the sensitivity of pneumococcal nasopharyngeal density was suboptimal, limiting its use as a diagnostic in a clinical setting [7]. In our study, only 39 cases had a pneumococcal density higher than this cutoff and very few children had a radiograph performed, so we were unable to perform analysis using this cutoff or any other pneumonia definition due to the limited number of cases. Further studies determining the relationship between nasopharyngeal carriage and lung or pleural aspirates would assist in determining the etiology of pneumonia and help to determine the sensitivity and specificity of pneumococcal colonization density as a surrogate for pneumococcal pneumonia for PCV impact studies.

However, the value of nasopharyngeal pneumococcal density for epidemiological purposes needs further exploration. Our findings suggest a dose-dependent association; that is, the probability of having severe pneumonia increased as the pneumococcal nasopharyngeal density increased. As many LMICs are unable to perform radiographs and do not have adequate microbiology services, finding an association between pneumococcal density and the current (2013) WHO severe pneumonia definition is important for these settings and should be further explored. Pneumococcal density could be an additional way to measure the impact of PCV for epidemiological purposes.

There were a number of limitations to our study. First, the allocation of participants to the severe pneumonia or other ARI group was based on a review of clinical findings, which may have varied between clinicians and led to misclassification. However, pediatricians in Mahosot Hospital are taught to classify pneumonia according to the 2013 WHO severe pneumonia definition, so this should have been minimized. Secondly, only 1 nasopharyngeal swab on admission was collected per child. Recent research has found that pneumococcal density may vary throughout a colonization episode [27]. Therefore, taking only a single nasopharyngeal swab may not capture peak pneumococcal density. However, in our study, duration of illness was not associated with severity: median was 3 days (IQR, 2–5 days) among severe, and 4 days (IQR, 2–5 days) in other ARI (*P* = .922), so duration of illness is unlikely to be a confounder. We found that there was no difference between the duration of illness between severe pneumonia and other ARI cases prior to admission; therefore, it is likely that the swab was taken at a similar time from the onset of symptoms for the severe pneumonia and other ARI groups. Third, children with pneumococci in the nasopharynx who are coinfected with respiratory viruses tend to have high nasopharyngeal pneumococcal densities [8, 10, 28]. In our study, we used the RSV data in the regression model and we adjusted for RSV as it is a potential confounder. During this observation period we also tested for influenza but only detected 33 cases. Influenza may affect density but as there were so few cases, we did not adjust for influenza in the analysis as it is very unlikely to have affected our results as influenza was rare. Finally, as there were missing data, we could not run the regression model on the whole data set. However, we found no major differences between those with complete and missing data, suggesting no systematic differences between the 2 groups. Our study is strengthened by using highly sensitive and specific laboratory techniques, and the fact that our findings are consistent with studies that have used more specific pneumococcal pneumonia definitions.

Determining the etiology of pneumonia and effect of PCV on pneumonia is very challenging as there is no case definition that is sensitive and specific enough to capture most cases. We have found pneumococcal colonization density to be a predictor of severe pneumonia in pneumococcal carriers in our study setting. Although causality cannot be ascribed due to the study’s observational design, further studies are warranted to determine whether this association exists in other settings, as this could be a potential outcome to measure the impact of PCV implementation for epidemiological purposes. Our findings contribute to a growing body of evidence demonstrating the positive association between pneumococcal density and severe pneumonia, and provide insight in the understanding of pneumococcal colonization density and the role it plays in pneumococcal disease.

## Supplementary Material

jiab239_suppl_Supplementary_Figure_S1

jiab239_suppl_Supplementary_Materials

## Notes

***Author contributions.*** O. J. J. C. and F. M. R. had full access to all the data in the study and take responsibility for the integrity of the data and the accuracy of the data analysis.

***Acknowledgments.*** We would like to thank the patients and their families for participation in this study. We are very grateful to the directors and staff of Mahosot Hospital; the late Dr Rattanaphone Phetsouvanh; and Dr Manivanh Vongsouvath, directors of the Microbiology Laboratory, the staff of the laboratory and the Lao-Oxford-Mahosot Hospital–Wellcome Trust Research Unit (LOMWRU), particularly the ARIVI team, for their technical help and support. We thank Associate Professor Chanphomma Vongsamphan, the Former Director of Department of Health Care, Ministry of Health, and Associate Professor Bounkong Syhavong, Minister of Health, Lao PDR, for their support for the work of LOMWRU. We also thank all the study, laboratory, and administrative staff at the Centre for International Child Health, Department of Paediatrics, University of Melbourne, Australia; Murdoch Children’s Research Institute, Australia; and the Expanded Programme on Immunization, Ministry of Health Lao PDR, including the late Dr Anonh Xeuatvongsa at the national immunization program. RSV testing was funded by the Institute of Research for Development, Aix-Marseille University, Wellcome Trust of Great Britain.

***Financial support.*** This analysis was part of a study funded by the Bill & Melinda Gates Foundation (grant number OPP1115490). F. M. R. is supported by National Health and Medical Research Council (NHMRC) fellowships. P. N. N. and D. A. B. D. are supported by the Wellcome Trust, which also provides core funds to LOMWRU. C. S. was supported by an Australian NHMRC Career Development Fellowship (award number 1087957) and a Veski Inspiring Women Fellowship.

***Potential conflicts of interest.*** C. N., C. S., E. D. M., and L. A. H. D. are investigators on a grant funded by Pfizer, outside the submitted work. All other authors report no potential conflicts of interest.
