# Supplementary figures and images for: Nasopharyngeal Pneumococcal Colonization Density Is Associated With Severe Pneumonia in Young Children in the Lao People’s Democratic Republic
Source: J Infect Dis. 2021 May 11;225(7):1266–73. doi: 10.1093/infdis/jiab239 (PMC8974848; doi:10.1093/infdis/jiab239)

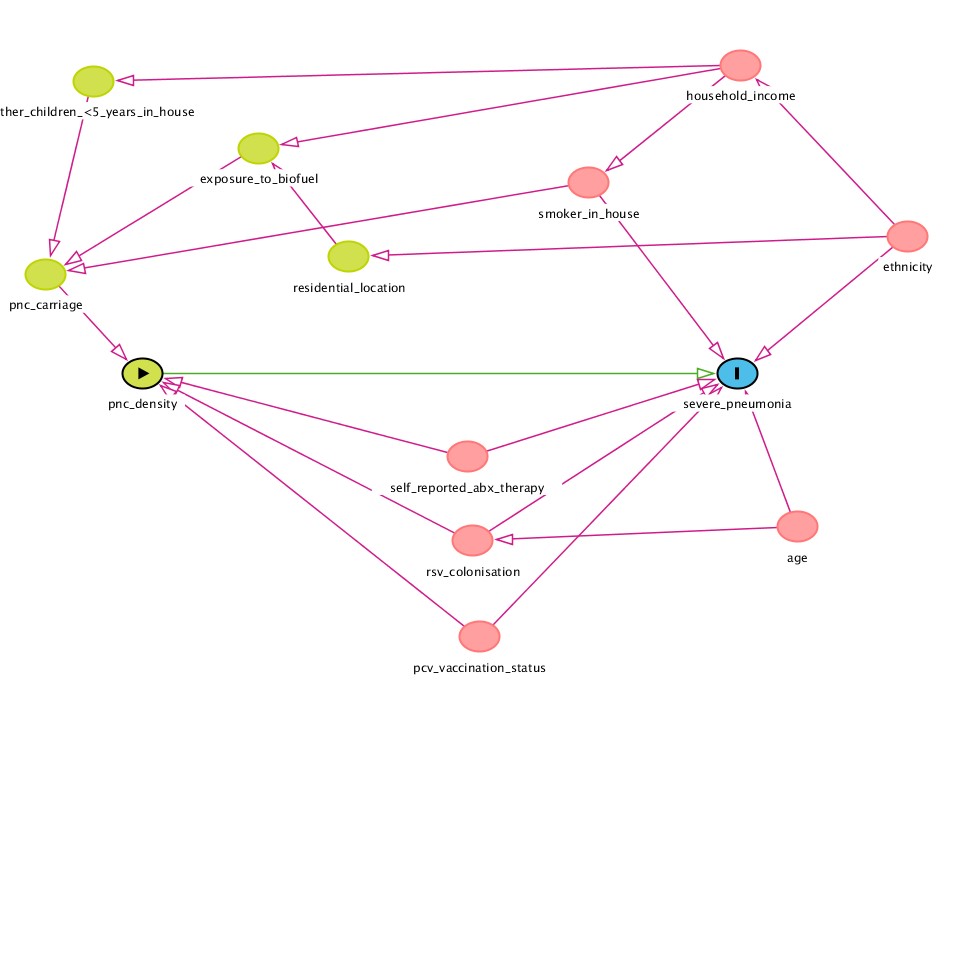

Supplement: jiab239_suppl_Supplementary_Figure_S1 [file jiab239_suppl_supplementary_figure_s1.jpeg]
