# Supplementary material for: Nasopharyngeal Pneumococcal Colonization Density Is Associated With Severe Pneumonia in Young Children in the Lao People’s Democratic Republic
Source: J Infect Dis. 2021 May 11;225(7):1266–73. doi: 10.1093/infdis/jiab239 (PMC8974848; doi:10.1093/infdis/jiab239)
Supplement: jiab239_suppl_Supplementary_Materials [file jiab239_suppl_supplementary_materials.docx]

**Supplementary Figures:**

Supplementary Figure 1: Directed acyclic graph depicting variables potentially associated with severe pneumonia^a^.

^a^ Software used was <http://www.dagitty.net/>. Blue is the outcome variable. Green with an arrow is the main exposure variable. Green with no arrows are ancestors of the exposure variable. Red are the ancestors of the exposure and outcome variable. Unidirectional arrows indicate causal relationships.

**Supplementary** **Tables:**

Supplementary Table 1: Comparison of pneumococcal carriers (n=468) included in the regression model for complete cases (n=372) versus the incomplete cases not included in the regression model (n=96)

| **Characteristics** | **All (n=468)** | **Complete cases (n=372)** | **Incomplete cases (n=96)** |
| --- | --- | --- | --- |
| **Main Variable** |  |  |  |
| Pneumococcal density^a^, median (IQR) | 5.7 (5.0 – 6.3) | 5.7 (5.0 – 6.3) | 5.6 (4.9 – 6.2) |
| **Demographics** |  |  |  |
| Age (months), median (IQR) | 14.7 (8.3 – 25.0) | 14.6 (8.6 – 24.9) | 15.1 (7.3 – 24.9) |
| Ethnicity, n (%) |  |  |  |
| Minority groups | 52 (11.1) | 39 (10.5) | 13 (13.5) |
| Lao Loum | 416 (88.9) | 333 (89.5) | 83 (86.5) |
| **Household features, n (%)** |  |  |  |
| Residential location |  |  |  |
| Outside Vientiane capital | 451 (96.3) | 365 (98.1) | 86 (89.6) |
| Other children <5 years old in house |  |  | n=94 |
| ≥2 | 176 (37.7) | 141 (37.9) | 35 (37.2) |
| Cigarette smoker in the house |  |  | n=89 |
|  | 207 (44.9) | 160 (43.0) | 47 (52.8) |
| Poverty line^b^ |  |  | n=91 |
| On/above poverty line | 429 (92.7) | 340 (91.4) | 89 (97.8) |
| **Clinical features** |  |  |  |
| Severity of pneumonia^c^ |  |  |  |
| Severe | 125 (26.7) | 94 (25.3) | 31 (32.3) |
| Non-severe | 343 (73.3) | 278 (74.7) | 65 (67.7) |
| RSV detection, n (%) |  |  | n=92 |
|  | 135 (29.1) | 110 (29.8) | 24 (26.1) |
| Prior antibiotic use^d^, n (%) | 212 (47.0) | 180 (48.4) | 32 (40.5) |

Abbreviations: PCV, pneumococcal conjugate vaccine; RSV, respiratory syncytial virus; IQR, interquartile range.

^a^ Pneumococcal density measured in log_10_ genome equivalents/ml.

^b^ Poverty line (World Bank) was defined as <USD1.25 per day (2013-2014); <USD1.9 per day (2015-2018) [[22](#_ENREF_22)].

^c^ World Health Organization (WHO). Pocket book of hospital care for children: guidelines for the management of common illnesses with limited resources.

^d^ Self-reported antibiotic therapy in the week before admission.
